# Triple-Negative Metaplastic Breast Cancer Ameliorated Following KEYNOTE-522: A Case Report

**DOI:** 10.7759/cureus.88751

**Published:** 2025-07-25

**Authors:** Edoardo G Frezza, Gayatri D Nimmagadda, Olutayo Sogunro

**Affiliations:** 1 Medicine, Trinity School of Medicine, Baltimore, USA; 2 Hematology and Oncology, Johns Hopkins Howard Hospital, Glen Burnie, USA; 3 Surgical Oncology, Johns Hopkins University School of Medicine, Baltimore, USA

**Keywords:** breast cancer, cardiac sarcoidosis (cs), keynote 522, metastatic metaplastic breast cancer, triple-negative breast carcinoma

## Abstract

Metaplastic breast cancer (MpBC) is a rare and aggressive subtype of breast cancer, with the triple-negative variant (TN-MpBC) being particularly resistant to standard systemic therapies and associated with poor outcomes. We present the case of a 48-year-old African American female diagnosed with TN-MpBC, incidentally identified during cardiac evaluation for sarcoidosis. Imaging and biopsy revealed a 5.8 cm high-grade tumor with a Ki-67 index above 30%. The patient was treated with the KEYNOTE-522 regimen, which includes neoadjuvant chemotherapy (NAC) (paclitaxel, carboplatin, doxorubicin, and cyclophosphamide) and pembrolizumab immunotherapy. Post-treatment imaging demonstrated substantial tumor regression, and subsequent bilateral mastectomy confirmed a complete pathological response with no residual malignancy. She is currently undergoing adjuvant pembrolizumab and proton radiation therapy. Given the historically poor response of TN-MpBC to chemotherapy alone, this case illustrates the promising role of immunotherapy, particularly in programmed death-ligand 1 (PD-L1)-expressing tumors. It supports emerging evidence that chemoimmunotherapy combinations such as KEYNOTE-522 may improve prognosis in TN-MpBC, emphasizing the need for continued investigation into targeted therapeutic strategies for this challenging breast cancer subtype.

## Introduction

Metaplastic breast cancer (MpBC) is a rare and biologically aggressive subtype, comprising less than 1% of all breast cancers [1]. It is histologically distinct from other breast carcinomas due to its mixed epithelial and mesenchymal differentiation and often presents with larger tumor size, higher grade, and more advanced stage at diagnosis [2,3]. A significant proportion of MpBCs fall under the triple-negative category (TN-MpBC), characterized by the absence of estrogen receptor (ER), progesterone receptor (PR), and human epidermal growth factor receptor (HER2/neu) expression, which limits targeted treatment options [1,4].

TN-MpBCs are often less responsive to standard neoadjuvant chemotherapy (NAC) compared to other triple-negative breast cancers (TNBCs), resulting in poor prognoses and higher recurrence rates [5,6]. Due to its rarity and histologic complexity, MpBC is frequently excluded from clinical trials, leaving treatment decisions to be guided by protocols designed for more common subtypes of TNBC. However, recent studies suggest that MpBCs may express programmed death-ligand 1 (PD-L1) and share molecular features with mesenchymal-like and immune-evasive breast cancer phenotypes, potentially making them candidates for immunotherapy [7].

This report presents a case of TN-MpBC in a 48-year-old African American female who demonstrated a complete pathological response to the KEYNOTE-522 regimen, which combines chemotherapy and pembrolizumab. This case underscores the emerging role of immune checkpoint inhibitors in managing rare and treatment-refractory breast cancers like TN-MpBC.

## Case presentation

The patient

A 48-year-old African American female with a history of cardiac sarcoidosis with cardiomyopathy presented for a cardiac PET scan. She had also incidentally noted a left breast mass. The PET scan further raised suspicion about the mass, prompting oncology evaluation. She reported a rapidly enlarging breast mass with discomfort. Physical examination revealed a large 5 cm, non-erythematous mass in the left breast with vague left axillary lymphadenopathy and no associated skin changes. Her family history was notable for a paternal first cousin with breast cancer in her early 40s.

Investigation

**Figure 1:**
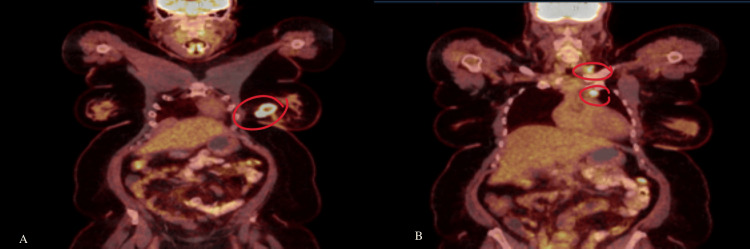
PET scan PET scan of the patient’s chest with a more exterior slice on the left and an interior slice on the right. A: highlights an increased FDG uptake in the left breast; B: shows an increased FDG uptake in a supraclavicular lymph node (2.1 SUV) and an infraclavicular lymph node (2.2 SUV). Both are circled in red. FDG: fluorodeoxyglucose; SUV: standardized uptake value

**Figure 2:**
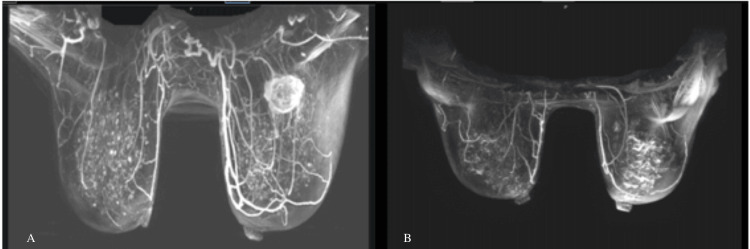
Breast MRI Breast MRIs before treatment (A) and after treatment (B). A: the heterogeneous mass in the left breast along the posterior breast tissue; B: a lack of mass.

**Figure 3:**
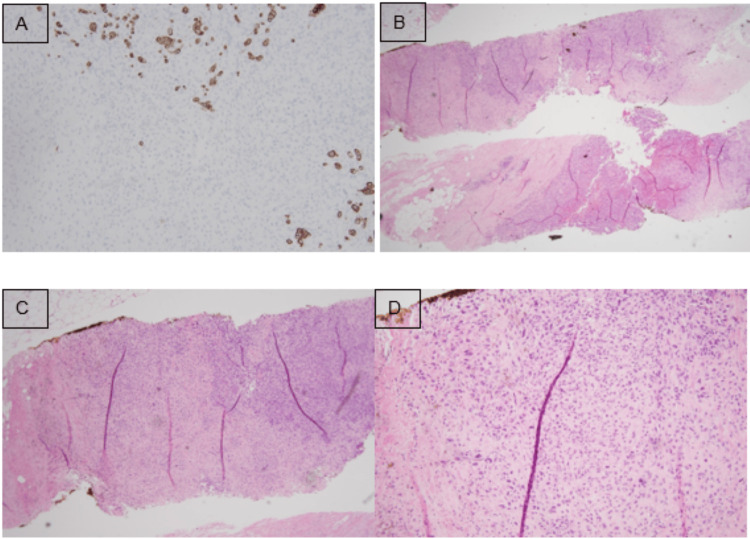
Histologic slides A: AE/AE3 stains showing some uptake of the cytokeratin markers in a portion of the biopsy. B: H&E slide shows a high-grade malignant neoplasm with two components on the slide. The top portion of epithelial marker (AE/AE3) positive carcinoma. The bottom portion of AE/AE3 negative high-grade sarcomatous-like malignant cells. C: Zoomed-in view of the upper portion of the H&E slide. A mix of AE/AE3 positive carcinoma and AE/AE3 negative sarcomatous-like malignant cells. D: Shows greater magnification of the same portion. There are areas of benign multinucleated giant cells. The malignant cells were negative for markers CD34, PAX8, TTF-1, and GATA3. Immunostaining of the biopsy revealed positive p63 stain in some malignant cells. The Ki-67 index was greater than 30%. The tissue sample was ER negative, PR negative, and HER2/neu negative. Her left axillary lymph node biopsy was negative for malignancy. ER: estrogen receptor; PR: progesterone receptor; HER2: human epidermal growth factor receptor

Her metabolic and hematological indices were unremarkable, and she tested negative for a multigene panel including BRCA 1 and 2. The workup included a PET scan identifying a 3.7 x 3.5 x 3.2 cm left breast mass with a standardized uptake value (SUV) of 4.4, with no evidence of distant metastasis and small supraclavicular/mediastinal lymph node uptake (Figure 1). A diagnostic mammogram and ultrasound revealed a 4.2 cm left breast mass, and an MRI (Figure 2) detected a 5.8 cm irregular mass with T2 hyperintensity extending to the pectoralis muscle, along with two smaller lesions (8 mm). A core needle biopsy of the breast mass confirmed triple-negative metaplastic carcinoma with Ki-67 >30% and negative axillary lymph node biopsy (Figure 3). The disease was staged as T3N0M0 (stage 3). The supraclavicular adenopathy was reviewed by a multidisciplinary team and thought to represent sequelae of her sarcoidosis. The patient declined an additional MRI-guided breast biopsy and instead opted for a bilateral mastectomy.

Treatment

She received NAC and neoadjuvant immunotherapy (NAI) as per the KEYNOTE-522 regimen: four cycles of paclitaxel, carboplatin, and pembrolizumab, followed by doxorubicin and cyclophosphamide with pembrolizumab for four cycles. An interim PET scan after two cycles showed a reduced tumor size (2.5 x 1.9 cm, SUV 1.1) and resolution of lymphadenopathy. After two cycles of doxorubicin, her ejection fraction fell to 40-45%, prompting a switch to liposomal doxorubicin, which restored her ejection fraction to 50%. Post-treatment MRI (Figure 2) revealed significant tumor reduction. The patient's neoadjuvant course lasted four months. She underwent a bilateral mastectomy. Pathological findings were a complete pathological response with no residual invasive carcinoma. She is now on adjuvant pembrolizumab for nine cycles. Due to the initial size of the mass being above 5 cm, she is also receiving proton radiation therapy for six weeks.

## Discussion

MpBC is an aggressive and histologically diverse form of breast cancer, characterized by neoplastic differentiation toward both epithelial and mesenchymal components [1,5]. Histologically, MpBC can be further categorized into subtypes such as spindle cell, squamous cell, and metaplastic carcinoma with mesenchymal differentiation, the latter being associated with the poorest prognosis [6]. These classifications primarily reflect the relative proportions of epithelial and mesenchymal components rather than distinct clinical subtypes. Clinically, MpBC often presents as a larger primary mass and is less likely to involve axillary lymph nodes compared to more common breast cancers [4]. Overall, MpBC carries a significantly worse prognosis than other subtypes such as invasive ductal carcinoma, infiltrating ductal carcinoma, and even triple-negative ductal carcinoma [8].

A notable feature of MpBC is its strong association with the triple-negative phenotype, marked by the absence of ER, PR, and HER2/neu expression [9]. TNBC is generally more aggressive and is linked to poorer prognoses than receptor-positive subtypes [9]. Although TNBCs can be sensitive to chemotherapy, response rates vary, and this inconsistency further limits effective treatment options for patients with TN-MpBC.

MPBCs, particularly their triple-negative variants, are known for their aggressive behavior and poor clinical outcomes. These tumors tend to metastasize earlier and are associated with lower survival rates than their non-metaplastic counterparts, including other forms of TNBCs [10]. Patients with TN-MpBC are also more likely to experience disease recurrence, and clinical data show that their tumors often respond poorly to conventional chemotherapy regimens. For instance, studies have reported significantly reduced rates of pathologic complete response (pCR) following NAC in this population [11,12]. These challenges underscore the urgent need for novel therapeutic strategies, such as the incorporation of immunotherapy, to improve outcomes in this difficult-to-treat breast cancer subtype.

Current treatment strategies for TNBC are influenced by the findings from the KEYNOTE-522 study. The study demonstrated that PD-L1 inhibitors provided improvement in overall survival in TNBC compared with chemotherapy alone [13]. Some studies suggest that MpBC expresses PD-L1 [7]. In their study, Joneja et al. identified that PD-L1 expression was detected in 46% of MpBC patients in their study (n=75), predominantly over other subtypes such as hormone-receptor positive and HER2/neu-receptor positive breast cancers [7]. Interestingly, PD-L1 expression was found in 9% of their TNBC samples (n=106). Furthermore, they also identified that the most common mutated genes in MpBCs were TP53 (56%) and PIK3CA (23%). These mutations may inform targeted immunotherapies in the future. Therefore, given the findings in our patient, Keytruda may be paramount for treating TN-MpBC. This aligns with other studies showing similar improvements [14,15].

The aggressive nature and limited responsiveness of TN-MpBCs to standard treatments highlight the need for further research in the development of effective treatment approaches. At the time of this case report, a recently published analysis of the SWOG1609 study demonstrated that the combination of cytotoxic T lymphocyte antigen 4 inhibitor ipilimumab and PD-1 inhibitor nivolumab showed exceptional response [16]. Three of seventeen patients responded well between 28 and 34 months, achieving pCR or partial responses. This study is encouraging, as it suggests a more specific treatment regimen for MpBC, but it is limited in understanding the exact mechanisms of action.

In comparison, there is a study that showed about 20/41 patients with MpBC had no response after approximately 35 months [5]. These patients received a doxorubicin, cyclophosphamide, and paclitaxel (ACT)-based NAC regimen, mostly with larger tumor sizes (median 2.4 cm) or advanced clinical T stage at presentation. Interestingly, this study showed that MpBC with predominant mesenchymal components responded to treatment compared to other subtypes. Within the same study, it is suggested that the molecular signature of MpBCs has similarities to the claudin-low and mesenchymal subtypes of TNBC. Whether or not this ultimately directs treatment protocols in the future is yet to be seen.

## Conclusions

This case underscores the potential of integrating immune checkpoint inhibitors into treatment regimens for TN-MpBC, a rare and aggressive subtype that typically demonstrates poor response to standard therapies. The patient’s complete pathological response following the KEYNOTE-522 regimen, combining NAC with pembrolizumab, highlights a promising therapeutic option for selected individuals with high-risk disease profiles. Clinically, this reinforces the relevance of incorporating immunotherapy early in the treatment course, particularly in patients whose tumors exhibit high proliferative indices or immune markers such as PD-L1. From a research perspective, this case contributes valuable real-world evidence supporting the role of chemoimmunotherapy in MpBC, while also emphasizing the need for expanded prospective studies and molecular profiling efforts to better define responsive subpopulations. Continued investigation into predictive biomarkers and histologic subtypes will be key to developing more tailored and effective treatment strategies for this underserved and understudied patient population.
